# Supplementary material for: Lessons Learned from Age-Friendly, Team-Based Training
Source: Geriatrics (Basel). 2023 Jul 28;8(4):78. doi: 10.3390/geriatrics8040078 (PMC10454452; doi:10.3390/geriatrics8040078)
Supplement: Supplementary file 1 [file geriatrics-08-00078-s001.zip › geriatrics-2247404-supplementary.pdf]

SUPPLEMENTARY GPLI PROGRAM AND COACHING INFORMATION

The goal of the Geriatric Practice Leadership Institute (GPLI) is to prepare teams of early and mid-career healthcare professionals to serve as leaders in their organizations based on the Age-Friendly (AF) Health Systems model developed by the Institute for Healthcare Improvement will not only benefit older patients and enrich leadership and organizational skills but also improve the delivery of care and financial outcomes of organizations. The suggested items and question prompts are collected from the IHI resources, and the structure is adopted from *Coaching as a Leadership Style* by Robert Hicks, Ph.D.

GPLI Curriculum Structure:

***3-week module length (content/assignments are estimated to be around 2 hours + coaching meeting and project work)**
Module Structure Recommendation:

week 1 = individual work
week 2 = teamwork
week 3= coach meeting and finalize work

Preparation for Coaching Sessions:

- Review of Module content- found on Canvas
- Coordination with the team on the virtual al meeting platform/time

Following Coaching Sessions:

- Communicate or connect any additional content expert support needed for the team
- Communicate any team challenges (i.e. access to Canvas, organization conflicts with team participation, team member issues, etc.) to GPLI Education Coordinator

Orientation:

Coaches Support for Thought

- Get to know your team (note: teams will be posting introductions on Canvas)
- Convey mutual respect/collegiality- equal roles as team members distinct from formal job hierarchy
    - Reinforce value to Strength Finders (SF)- identification and collaboration benefits;(more discussion of SF will occur during Module 1)

- Clarify understanding of project/value/team representatives
  - What problem do you plan to address in your healthcare setting?
  - How will it impact care delivery? (efficiency, safety, patient satisfaction, etc.)
  - What stakeholders will be impacted? (staff, patients, family/caregivers)
    - Are all represented on the team? If not, how will you gain insight from unrepresented stakeholders?
  - What barriers do you perceive with the delivery of your plan?
  - How do you plan to address barriers or challenges?
  - How will your innovation solve the problem addressed?
- Team Charter (initiate):
  - Contact information for coach + team members, sponsor
  - Meeting time opportunities/platform (Microsoft Teams, Zoom, etc.)
  - Expectations of coach and team

Module 1: The Iceberg of Culture & Strengths-Based Leadership

- **Competencies:**
  - Understand the importance of organizational culture and leadership in driving organizational change
  - Understand Kotter's eight stages of an organizational change
  - The pitfalls to avoid at each stage
  - How Kotter's model supports the implementation of your Age-Friendly project
  - Discuss the value of a strengths-based approach to leadership
  - Discuss and understand your assessment
  - Put in action what you have learned about your strengths and your role as a leader
- **Lecture:**
  - The Iceberg of Culture & Strengths Based Leadership
- **Readings:**
  - *Leading Change* (Kotter, 2012)
- **Assignment:**
  - StrengthFinder
  - Kotter's Planning for Change Template

Module 2: Leadership & Teams

- **Competencies:**
  - Better understanding of why engagement is so important
  - Learn how leadership style impacts motivation
  - Discover how important autonomy and purpose are to motivation and suggestions for improving both
  - Better understanding of the role of today's leaders
  - Learn how your values, beliefs, and operating principles impact your leadership
  - Understand more about how to enhance your impact as a leader as you engage people and lead change
- **Lecture:**
  - Lecture(s): Leadership Style and Motivation and Leading Self-Making and Impact
- **Readings:**
  - *HBR The Role of a Manager Has to Change in 5 Key Ways*
  - *How Are You Perceived at Work*
- **Assignment:**
  - Your Leadership Journey and How am I perceived at Work

Coaches Support for Thought

- How does your institution's current state compare to 4M's AF HC framework for your care innovation interest?
  - Are there gaps in the 4M's? How will you fill in the gaps?
  - What staff members are involved in addressing the 4M's? Are they represented on your team?
- What processes, tools, and resources do you already have to address the needs of the 4M's?
  - Are there gaps to address the needs of your innovation to address the 4M's?
  - How will you attain/improve the processes/tools/resources?
- How do you plan to gain buy-in and implement your plan within the larger institution?
  - Are there opportunities for prompts in the environment, EMR, protocols, etc., to promote your innovation?
  - How will you address these needs?
  - How will the innovation impact the broader healthcare team?
- How will your innovation positively impact older adults and family caregivers?
  - How will you measure this impact?

        - How will any provided resources address the health literacy level of older adults?
    - What community-based resources could benefit your team or innovation?

- Strengths Based Leadership
    - What are your strengths? Which findings strongly resonated with your own beliefs? (allow each team member to share)
    - How do you feel your strengths can provide leadership support to the efforts of the team innovation?
    - How do you feel your team member (allow them to select one- be sure all are accounted for) can use their strengths to contribute to a team need?

Module 3: Age-Friendly Health Systems Overview

- **Competencies:**
    - Describe and explain the 4Ms framework of the age-friendly model
    - Analyze current practice state
    - Understand how QI fits into the Age-Friendly Health System model
    - Describe the benefit to an organization that implements an age-friendly model
- **Lecture:**
    - Introduction to Age-Friendly
- **Readings/Videos:**

- What is an Age-Friendly Health System (Links to an external site.)
- Key Components of Age-Friendly Care (Links to an external site.)
- **Providing Optimal Care for Older Adults** (Links to an external site.)
- **Age-Friendly Health Systems Interview with Dr. Terry Fulmer** (Links to an external site.)

- **Assignment:**
    - Health Systems Background Overview

Coaches Support/Challenge for Action/Challenge for Thought

- AF Healthcare Design/description (*AF Appendix C: 4Ms AF Care Description Worksheet*)
    - What are the **Aims** for each 4M's as they contribute to your innovation?

        - How will you **Engage/Screen/Assess** the implementation of each of the 4M's?
        - How often or when (**frequency**) will attention be provided for your plan?
        - How will **documentation** of these aims occur to assess outcomes?
        - How will you **act on** the 4M's to impact quality improvement in these areas?
        - Who will have the **primary responsibility** to improve these actions?

  - Test Key Actions
    - How do your key actions address each of the 4Ms? (Appendix D: Key Actions and Getting Started with AF Care)
    - How will your team members be involved in supporting implementation/documentation? (*Appendix E: AF Care Workflow examples)*
    - How will this workflow be monitored? (*Appendix F: Example PDSA Cycles for AF Care*)
    - How will you adapt your workflow to meet communicated/observed challenges?

Module 4: Quality Improvement in an Age-Friendly Health System

- **Competencies:**
  - Understand how QI fits into the Age-Friendly Health System model
  - Participants will understand how the PDSA cycle can be integrated with the 4Ms
  - Document and discuss PDSA implementation
- **Lecture:**
  - Quality improvement in an age-friendly system
- **Readings/Videos:**
  - *IHI Quality Improvement Essentials Toolkit*
  - PDSA Part 1 (Links to an external site.)
  - PDSA Part 2 (Links to an external site.)
  - From CLABSIs to Cucumbers (Links to an external site.)
  - Flowcharts
- **Assignment:**
  - Project Charter

Coaches Challenge for Action/Challenge for Thought

  - Implementing Care

        - How will you support your team and expanded healthcare force in implementing your AF innovation?
        - How will the healthcare team be prompted to engage in delivery?
    - Studying performance
        - How do you plan to collect data to measure the impact of your 4Ms innovation?
        - How will your plan improve the financial outcomes of your organization? (see IHI AFHS Guide to 4Ms, Appendix H: *Measuring the Impact of 4Ms AF Care or* Appendix G: *Implementing Reliable 4Ms Age-Friendly Care)*
        - How will data be used to implement RCQI adjustments, if needed? (See: IHI AFHS Guide to 4Ms, Appendix F: *Example PDSA Cycles for AF Care)*

Module 5: Putting the 4Ms into Practice

- **Assignment:**
    - Age-friendly, quality improvement project

All learners will submit the Following Activities and Documents:

- Leadership Self-Assessment
- Age-Friendly Baseline Documentation
- Quality Improvement Project Charter
- Relevant Project Documentation
- Final Project Presentation

Coaches Challenge for Thought

- Sustaining Care
    - How will you know that the 4Ms care, as described in your plan, is reliably in place? (*Appendix G: Implementing Reliable 4Ms AF Care)*
    - How can you identify champions to extend the efforts of your team?
    - How will you address ongoing challenges/needs?
    - How often will you address the need for studying performance for potential improvements?

Coaches Support for Action

Project Delivery

- How is the progress of your AF project implementation?
- What roles have your team members accepted in delivery? Are additional staff involved?
- How has implementation been received by your facility/staff?
- How well has the innovation been implemented into current procedures?

PDSA application

- What have been unexpected outcomes or insights from your implementation process?
- Has your team faced challenges with implementation? If so, how did you address it?
- Have you applied and documented the PDSA process for addressing needs?

Outcome Measures

- What are some observable successes from the implementation thus far?
- How are you collecting data to demonstrate the impact of your project?
- Have you considered the value of quantitative and qualitative outcomes from all stakeholders?

Project Presentation

- How do you plan to share the 'project journey' and outcomes with your facility, community, and GPLI peers?
- Besides your team and sponsor, whom would you like to invite to the graduation to hear of your team's efforts and successes?
- How can the GPLI faculty and myself support your efforts to share your work and success story?
